# Type 2 and type 3 gastric neuroendocrine tumors have high risk of lymph node metastasis: Systematic review and meta‐analysis

**DOI:** 10.1111/den.15026

**Published:** 2025-04-01

**Authors:** Yohei Ogata, Waku Hatta, Takeshi Kanno, Masahiro Saito, Xiaoyi Jin, Naoki Asano, Tomoyuki Koike, Akira Imatani, Yuhong Yuan, Atsushi Masamune

**Affiliations:** ^1^ Division of Gastroenterology Tohoku University Graduate School of Medicine Miyagi Japan; ^2^ Department of Medicine London Health Science Centre London Ontario Canada

**Keywords:** gastric neuroendocrine tumor, lymph node metastasis, pathological risk factor, type 2

## Abstract

**Objectives:**

Lymph node metastasis (LNM) is crucial in determining treatment strategies for gastric neuroendocrine tumors (gNETs). While type 3 is considered more aggressive than types 1 and 2 within the clinical subtype of gNETs, the supporting data were insufficient, due to their rarity. We aimed to study the prevalence and risk factors associated with LNM in gNETs.

**Methods:**

We searched electronic databases from 1990 to 2023 to identify case–control and cohort studies regarding gNETs resected either endoscopically or surgically. The primary outcome measured was the pooled prevalence of LNM in gNETs. Secondary outcomes included categorizing the prevalence of LNM by clinical subtypes and identifying pathological risk factors associated with LNM in gNETs.

**Results:**

We included 28 studies, involving 1742 patients, among whom 240 had LNM (pooled prevalence rate, 11.8%; 95% confidence interval 7.6–17.9%). The pooled prevalence rates of LNM for type 1, type 2, and type 3 gNETs were 6.0%, 38.5%, and 23.2%, respectively. Type 2 (odds ratio [95% confidence interval] 11.53 [3.46–38.49]) and type 3 (6.88 [3.79–12.49]) gNETs exhibited a higher risk for LNM compared to type 1. Pathological risk factors for LNM included tumor size >10 mm (4.18 [1.91–9.17]), tumor invasion into the muscularis propria or deeper (11.21 [3.50–35.92]), grade 2/grade 3 (5.96 [2.65–13.40]), and lymphovascular invasion (34.50 [6.70–177.51]).

**Conclusion:**

We demonstrated that type 2 gNETs, as well as type 3, had a high risk of LNM. Additionally, four pathological risk factors associated with LNM were identified.

## INTRODUCTION

Neuroendocrine neoplasms are classified into well‐differentiated neuroendocrine tumors (NETs) and poorly differentiated neuroendocrine carcinomas (NECs), with the former conventionally referred to as “carcinoid.”^1^ NETs are characterized by a relatively indolent rate of growth compared to other epithelial malignancies; however, some of them can be aggressive and resistant to therapy.^2^, ^3^ Gastric NETs (gNETs) are rare, with an incidence of 0.15 per 100,000, accounting for ~6.9–8.7% of all digestive NETs^4^ and representing less than 2.0% of all gastric malignancies.^5^ However, with the widespread utilization of screening endoscopy, the incidence of gNETs is on the rise.^6^

Gastric NETs are divided into three clinical subtypes based on underlying diseases^7^, ^8^: type 1, the most common, arises from hyperplasia of enterochromaffin‐like (ECL) cells due to the hypergastrinemia caused due to chronic atrophic gastritis; type 2, histologically similar to type 1, is associated with Zollinger–Ellison syndrome; and type 3 arises sporadically and is independent of gastrin. Type 3 gNETs are generally considered more aggressive than type 1 and type 2^7^, ^8^; however, the prevalence rate of lymph node metastasis (LNM) in each subtype is not well‐described.

Lymph node metastasis serves as a critical prognostic factor in gNETs and plays a pivotal role in determining the treatment strategy, such as opting for endoscopic resection (ER) or surgical resection (SR), for these tumors. Moreover, besides the inadequate understanding of LNM prevalence based on clinical subtype, the pathological risk factors associated with LNM remain unknown, because prior reports included a small number of cases. Therefore, we conducted a systematic review and meta‐analysis of all available studies to elucidate the prevalence and risk factors associated with LNM in gNETs.

## Methods

### Search strategy for study identification

We searched the databases Medline, Embase, Cochrane Central Register of Controlled Trials, and Cochrane Database of Systematic Reviews, via OvidSP for eligible studies published between 1990 and June 9, 2023, with restrictions of human studies published in English. The search strategy was designed and performed by an experienced medical information specialist (Y.Y.) with input from the study's investigators. It employed controlled vocabulary supplemented with keywords to identify studies about gastrointestinal NETs. The complete search strategy is provided in Appendix S1. This systematic review and meta‐analysis adhere to the Preferred Reporting Items for Systematic Reviews and Meta‐Analyses (PRISMA) statement (Appendix S2).^9^ Our initial systematic review protocol was registered at the International Prospective Register of Systematic Reviews (PROSPERO registration number: CRD42020198582, https://www.crd.york.ac.uk/prospero/).

### Inclusion and exclusion criteria

This systematic review included case–control and cohort studies meeting the following criteria: (i) gNETs were resected either endoscopically or surgically; (ii) the sample size was ≥10 patients; (iii) LNM was evaluated; or (iv) risk factors associated with LNM were assessed. The exclusion criteria were as follows: (i) studies published in languages other than English; (ii) case series, case reports, review articles, letters to the editor, comments, editorials, and conference proceedings; and (iii) studies exclusively involving patients with NECs.

### Definition of LNM

Pathological confirmation of LNM was conducted in SR cases with lymph node dissection. Conversely, for cases involving ER or local resection, LNM was assessed clinically, including radiological examinations before treatment and throughout the follow‐up period.

### Study selection and data extraction

Two investigators, Y.O. and W.H., independently screened all titles and abstracts for gastrointestinal NETs to identify studies meeting the inclusion criteria. Subsequently, the two investigators independently extracted data. The predesigned data extraction sheet encompassed author information, country of origin, year of publication, study design, study population characteristics, cohort size, treatment approach, frequency of LNM, clinical subtype, pathological factors (tumor size, depth, World Health Organization [WHO] grading system,^10^ and lymphovascular invasion [LVI]), prognosis, and follow‐up duration. Any disagreements between investigators were resolved through discussion. If consensus was not reached between the two investigators, a third investigator (T.K.) was consulted for resolution.

### Outcomes assessment

The primary outcome of this systematic review was to determine the pooled prevalence rate of LNM in gNETs. Secondary outcomes included the prevalence rates of LNM classified according to the clinical subtype and the identification of pathological risk factors associated with LNM in gNETs. Candidate pathological risk factors included tumor size >10 mm, tumor invasion into the muscularis propria (MP) or deeper, grade 2 (G2)/grade 3 (G3), and the presence of LVI.

### Quality assessment

Two investigators (Y.O. and W.H.) independently assessed the risk of bias using the Joanna Briggs Institute (JBI) Critical Appraisal Tools for use in JBI Systematic Reviews for prevalence studies.^11^ The risk of bias was classified as high, moderate, or low based on the study's percentage score: ≤49% for high, 50–69% for moderate, and ≥70% for low, each indicating a “yes” score. Any discrepancies between the two investigators were resolved through discussion. If necessary, resolution was achieved by having a third investigator (T.K.) independently review the corresponding study.

### Data synthesis and statistical analysis

The Freeman–Tukey double arcsine method and inverse variance method were utilized for calculating the pooled proportion and corresponding 95% confidence interval (CI) of prevalence. The pooled odds ratio (OR) and its 95% CI for the association of risk factors and LNM were calculated using the Mantel–Haenszel random effects model.^12^ When available, raw data were recorded, and the OR was calculated based on the provided raw data. The inverse variance method was employed to pool the ratios. We utilized a random effects model in all meta‐analyses,^13^ to ensure a more conservative estimate of the effect.

We assessed statistical heterogeneity among studies using Cochran's Q test (significant at *P* < 0.10) and quantified it with the inconsistency index (*I* ^2^) statistics.^14^ *I* ^*2*^ statistics values of <30%, 30–59%, 60–75%, and >75% were categorized as indicating low, moderate, substantial, and considerable heterogeneity, respectively. Subgroup analyses were conducted to investigate between‐study sources of heterogeneity. In the subgroup analysis, a *P*‐value for the difference between subgroups (*P*_interaction_) of <0.10 was deemed statistically significant. Analyses were performed for geographic area, clinical subtype, treatment method, and study quality. We assessed publication bias qualitatively using the funnel plots and quantitatively using Egger's test,^15^, ^16^ when there were ≥10 studies in the meta‐analysis, consistent with previous recommendations.^17^ If the *P*‐value was ≤0.10 in this test, we considered publication bias to be present. Statistical analyses were conducted using R version 4.2.1 (R Foundation, Vienna, Austria).

## RESULTS

### Search and selection result

**Figure 1:**
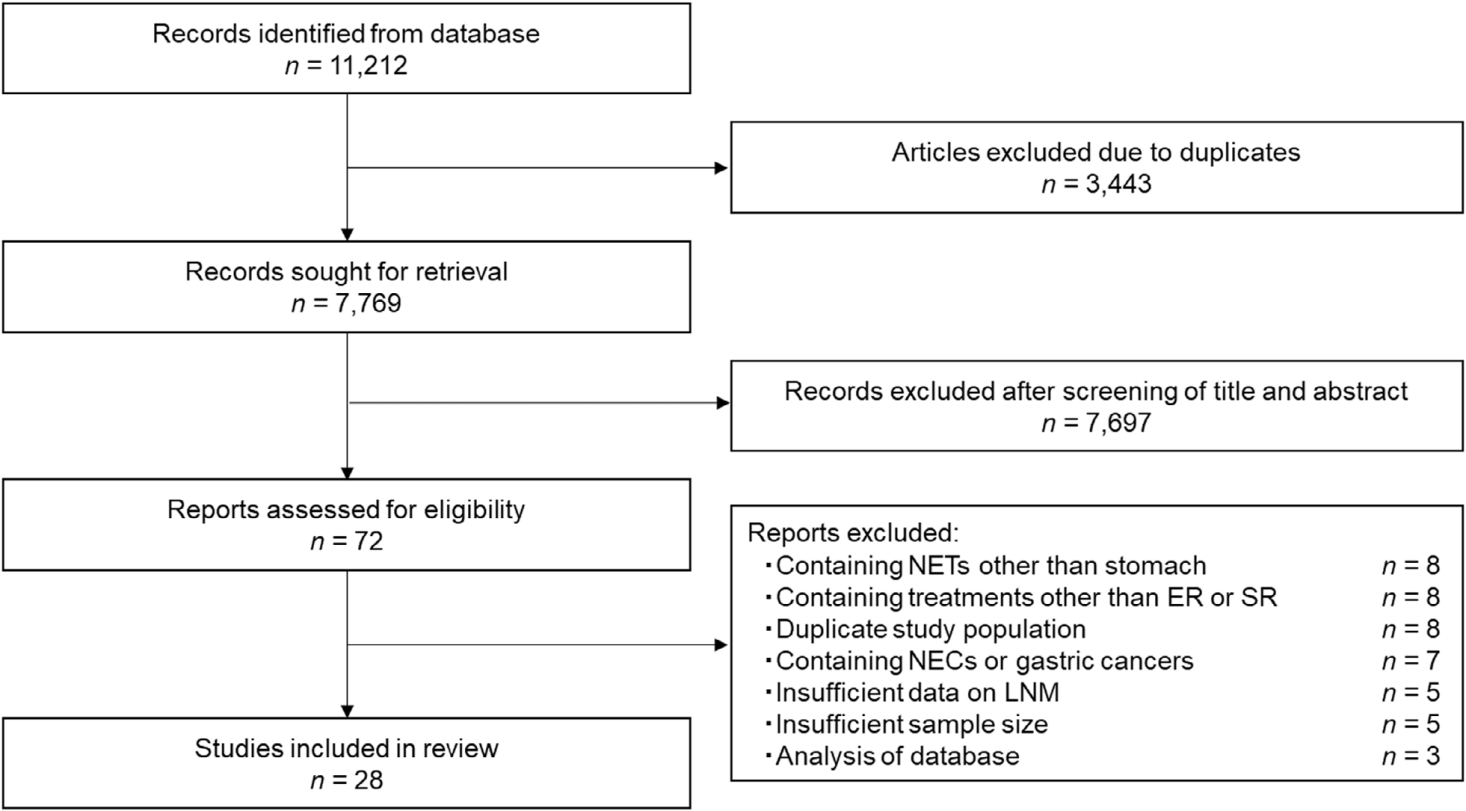
PRISMA flow diagram of the study selection process. ER, endoscopic resection; LNM, lymph node metastasis; NEC, neuroendocrine carcinoma; NET, neuroendocrine tumor; SR, surgical resection.

A total of 11,212 articles on gastrointestinal NETs were identified from the database. After excluding 3443 articles due to duplicates, we screened 7769 articles based on the titles and abstracts. Subsequently, we excluded 7697 articles for various reasons; the main reason for exclusion was NETs other than the stomach. Then we evaluated 72 full‐text articles for eligibility. Ultimately, 28 articles^4^, ^18^, ^19^, ^20^, ^21^, ^22^, ^23^, ^24^, ^25^, ^26^, ^27^, ^28^, ^29^, ^30^, ^31^, ^32^, ^33^, ^34^, ^35^, ^36^, ^37^, ^38^, ^39^, ^40^, ^41^, ^42^, ^43^, ^44^ were eligible for inclusion after exclusion of 44 articles (eight articles, containing NETs other than stomach; eight articles, containing treatments other than ER or SR; eight articles, duplicate study population; seven articles, containing NECs or gastric cancers; five articles, including insufficient data on LNM; five articles, insufficient sample size; three articles, analysis of the database). The PRISMA flow diagram of the study selection process is presented in Figure 1.

### Characteristics and quality of included studies

The characteristics of eligible studies are provided in Table 1 and Table S1. Seventeen were from Asia,^4^, ^20^, ^22^, ^23^, ^24^, ^25^, ^28^, ^29^, ^30^, ^34^, ^35^, ^37^, ^38^, ^40^, ^41^, ^42^, ^43^, ^44^ eight were from Europe,^18^, ^19^, ^21^, ^27^, ^31^, ^32^, ^37^, ^39^ and three were from the United States.^26^, ^33^, ^36^ Thirteen were multicenter studies, and 15 were single‐center studies.

**Table 1:** Major characteristics of eligible studies about gastric neuroendocrine tumors

Authors	Year	Country	Enrollment time period	Type of treatment (*n*)	No. of patients	LNM in SR cases	LNM in all cases
Rindi *et al*.	1995	Italy	NA	ER (12), SR (43)^†^	45	NA	4
Li *et al*.	2012	China	2008–2010	ER (18), SR (1)^‡^	19	0	0
Merola *et al*.	2012	Italy	1993–2008	ER (32), SR (1)^‡^	33	0	0
Endo *et al*.	2012	Japan	1998–2011	ER (3), SR (13)	16^§^	2	2
Uygun *et al*.	2014	Turkey	1999–2012	ER (22)	22	NA	0
Jung *et al*.	2015	Korea	1996–2010	ER (18), SR (8)	26	0	0
Shen *et al*.	2016	China	2009–2015	ER (25), SR (110)^†^	46	NA	7
Lee *et al*.	2016	United States	1994–2015	ER (16), SR (29)	45	11	11
Sagatun *et al*.	2016	Norway	1993–2013	SR (8)	8^§^	3	3
Xu *et al*.	2016	China	1995–2015	SR (3)^¶^	3	0	0
Chung *et al*.	2018	Taiwan	2010–2016	ER (57), SR (55), Unknown (75)	187	NA	24
Min *et al*.	2018	Korea	2005–2014	ER (17), SR (15)^‡^	32	2	3
Vanoli *et al*.	2018	Italy	1980–2017	ER (87), SR (70)	157	NA	16
Daskalakis *et al*.	2019	Greece	1997–2017	ER (97), SR (17)	114	NA	3
Crown *et al*.	2019	United States	2000–2016	ER (25), SR (50)	75	NA	12
Chen *et al*.	2019	China	2011–2018	ER (24)	24	NA	0
Chen *et al*.	2020	China	2012–2019	ER (136)	136	NA	0
Trinh *et al*.	2020	United States	2002–2019	SR (66)	66	12	12
Chin *et al*.	2021	Ireland	2005–2017	ER (12), SR (7)	19^§^	NA	3
Exarchou *et al*.	2021	UK	2006–2019	ER (10), SR (26)	36^§^	11	11
Hirasawa *et al*.	2021	Japan	1987–2015	ER (48), SR (96)^‡^	144	15	15
Li *et al*.	2021	China	2012–2019	SR (94)	94	68	68
Exarchou *et al*.	2022	UK	2003–2019	ER (10), SR (8)	18^§^	1	1
Kurtulan *et al*.	2022	Turkey	2000–2015	SR (44)	44	15	15
Ryu *et al*.	2022	Korea	2008–2020	ER (14), SR (4)	18^§^	2	2
Sekar *et al*.	2022	India	2011–2020	Unknown (65)	65	NA	18
Kim *et al*.	2023	Korea	2000–2020	ER (116), SR (23)	139	6	6
Namikawa *et al*.	2023	Japan	1991–2019	ER (84), SR (27)^‡^	111^§^	4	4

^†^ Including 99 cases with neuroendocrine carcinomas, mixed neuroendocrine–nonneuroendocrine neoplasia, and gastrinomas in the number of treatments, which were excluded from this meta‐analysis; 10 in the report by Rindi *et al*. and 89 in the report by Shen *et al*.

^‡^ Including 25 cases with additional gastrectomy; one in the report by Li *et al*., one in the report by Merola *et al*., five in the report by Min *et al*., 15 in the report by Hirasawa *et al*., and three in the report by Namikawa *et al*.

^§^ There were some cases with other treatments in each report, and such cases were not included in this meta‐analysis; three cases with no treatment in the report by Endo *et al*., 18 cases with no resection by Sagatun *et al*., 30 cases with no treatment by Chin *et al*., nine cases with no resection by Exarchou *et al*. (2021), 87 cases with no treatment by Exarchou *et al*. (2022), four cases with no treatment by Ryu *et al*., 61 cases with no treatment by Namikawa *et al*.

^¶^ Only patients with type 2 gastric neuroendocrine tumors were included in the analysis, given the clearly stated type of treatment.

ER, endoscopic resection; LNM, lymph node metastasis; NA, not applicable; SR, surgical resection.

The quality assessment of the included studies based on the JBI Critical Appraisal Tools for use in JBI Systematic Reviews is provided in Table S2. Twenty‐four studies were classed as being at low risk, and four were at moderate risk. There was no study on high risk.

### Pooled prevalence rate of LNM in gNETs

**Figure 2:**
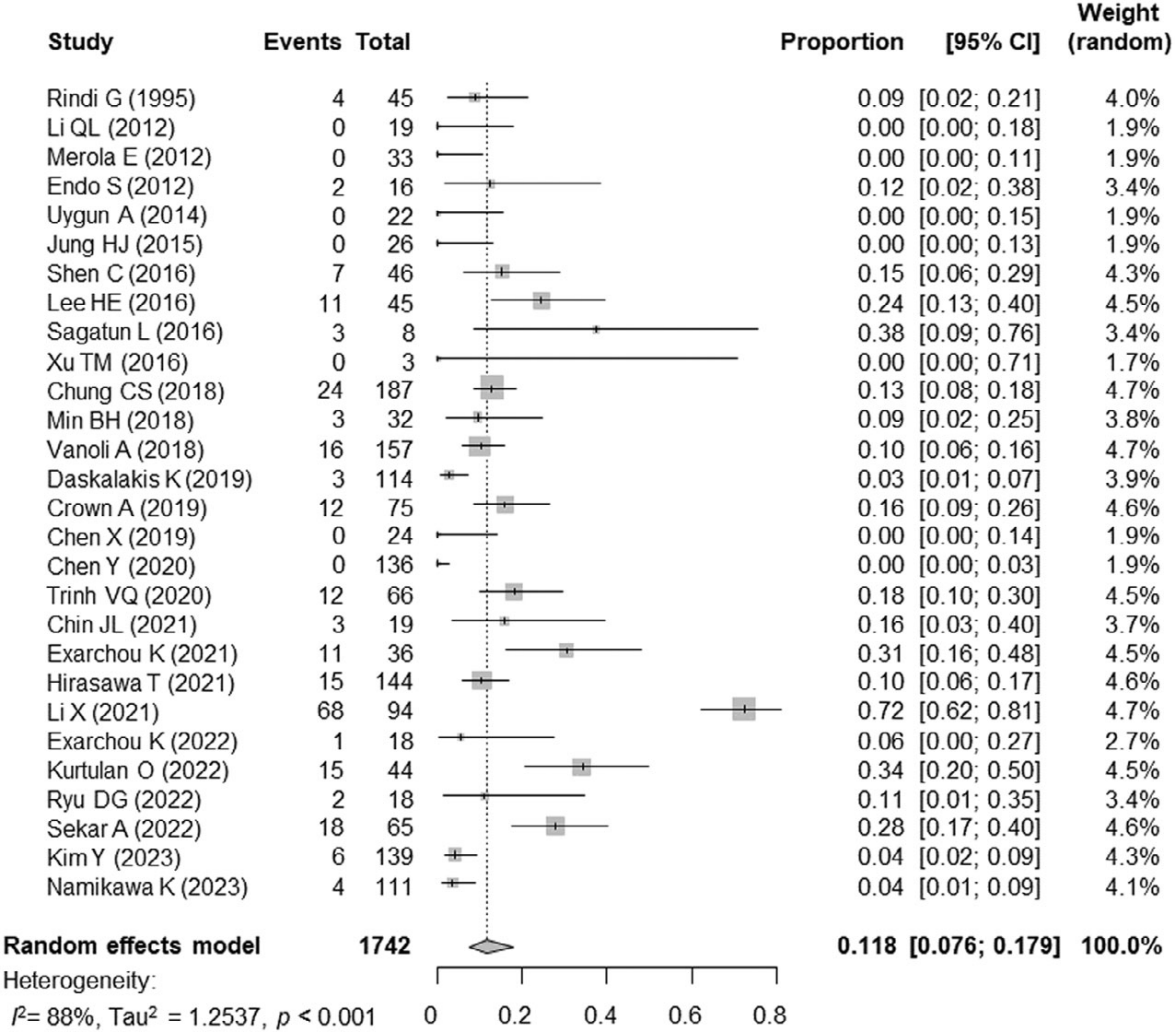
Forest plot of pooled prevalence rate of lymph node metastasis in gastric neuroendocrine tumors. CI, confidence interval.

When we pooled data from 28 studies in 1742 patients with gNETs, there were 240 patients with LNM. The pooled prevalence rate of LNM in gNETs was 11.8% (95% CI 7.6–17.9%), with considerable heterogeneity across studies (*I* ^*2*^ = 88%) (Fig. 2). In exploring potential sources of heterogeneity, a significant difference in the prevalence of LNM was observed among clinical subtypes (*P*_interaction_ <0.001) and between ER and SR (*P*_interaction_ <0.001). It should be noted, however, that 524 and 708 cases, respectively, were excluded from the subgroup analyses due to the lack of information (Table 2). Conversely, no significant difference was observed among the geographic areas (*P*_interaction_ = 0.664) or between the study qualities (*P*_interaction_ = 0.717).

**Table 2:** Subgroup analyses of pooled prevalence rates of lymph node metastasis (LNM) in gastric neuroendocrine tumors

	No. of studies	No. of cases	No. of LNM	Pooled prevalence, % (95% CI)	*P*_interaction_
Overall	28	1742	240	11.8 (7.6–17.9)	–
Geographic area					0.664
Asia	17	1126	164	10.2 (5.1–19.4)	–
Europe	8	430	41	11.2 (5.6–21.1)	–
United States	3	186	35	19.0 (14.0–25.4)	–
Clinical subtype^†^					<0.001
Type 1	15	713	29	6.0 (3.7–9.5)	–
Type 2	5	28	11	38.5 (19.0–62.6)	–
Type 3	14	477	92	23.2 (16.0–32.3)	–
Treatment method^‡^					<0.001
ER	15	568	1^§^	2.2 (1.1–4.4)	–
SR	17	466	152	26.9 (17.0–39.7)	–
Study quality (risk of bias)					0.717
Low	24	1391	186	11.0 (6.4–18.3)	–
Moderate	4	351	54	16.4 (9.5–26.9)	–

^†^ 524 cases in seven studies were excluded due to the unknown clinical subtype.

^‡^ 708 cases in eight studies were excluded because they included endoscopic resection (ER) and surgical resection (SR) cases, but lacked information on the number of cases and/or LNM cases in each of ER and SR.

^§^ Only one case with 1.6 cm grade 1 neuroendocrine tumor reported by Min *et al*. (2018) developed perigastric LNM 68 months after ER.

CI, confidence interval.

### Prevalence of LNM according to the clinical subtype

Seventeen studies reported the clinical subtype. The pooled prevalence rates of LNM in type 1, type 2, and type 3 gNETs were 6.0% (95% CI 3.7–9.5%), 38.5% (95% CI 19.0–62.6%), and 23.2% (95% CI 16.0–32.3%), respectively, with moderate heterogeneity in type 1 (*I* ^*2*^ = 38%) and substantial heterogeneity in type 3 (*I* ^*2*^ = 67%) (Table 3). When cases were limited to those with SR as a sensitivity analysis, the pooled prevalence rates of LNM remained higher in type 2 (26.8%; 95% CI 7.8–61.5%) and type 3 (29.1%; 95% CI 17.8–43.7%) compared to type 1 (16.4%; 95% CI 9.4–26.9%), with substantial heterogeneity in type 3 (*I* ^*2*^ = 63%) (Table 3). When one study with a notably high rate of LNM (64.3%) in type 3 gNETs^40^ was excluded, the prevalence rate of LNM in type 3 gNETs with SR was 23.7% (95% CI 15.6–34.1%), with no significant heterogeneity.

**Table 3:** Pooled prevalence rates and sensitivity analysis of lymph node metastasis (LNM) in gastric neuroendocrine tumors according to the clinical subtype

	No. of studies	No. of cases	No. of LNM	Pooled prevalence, % (95% CI)	*P* for Q test	*I* ^*2*^	*P* for Egger's test^†^
Clinical subtype							
Type 1	15	713	29	6.0 (3.7–9.5)	0.065	38	0.192
Type 2	5	28	11	38.5 (19.0–62.6)	0.260	24	–
Type 3	14	477	92	23.2 (16.0–32.3)	<0.001	67	0.932
Sensitivity analysis with SR cases^‡^						
Type 1	4	72	11	16.4 (9.4–26.9)	0.810	0	–
Type 2	2	9	2	26.8 (7.8–61.5)	0.472	0	–
Type 3	9	229	53	29.1 (17.8–43.7)	0.006	63	–
Sensitivity analysis with SR cases excluding outlier study^§^							
Type 3	8	215	44	23.7 (15.6–34.1)	0.127	38	–

^†^ Egger's test was not performed in the analysis due to the number of studies being <10.

^‡^ Studies that included endoscopic resection (ER) and surgical resection (SR) cases but lacked information on the number of cases and/or LNM cases in each of ER and SR were excluded from this analysis.

^§^ The study by Kurtulan *et al*., with an LNM prevalence rate of 64.3% when limited to type 3, was further excluded.

CI, confidence interval.

There was a significantly higher risk for LNM in type 2 (OR 11.53; 95% CI 3.46–38.49), and type 3 (OR 6.88; 95% CI 3.79–12.49) gNETs than in type 1, whereas the LNM risk did not significantly differ between type 2 and type 3 gNETs (Table 4).

**Table 4:** League table for risk of lymph node metastasis in gastric neuroendocrine tumors according to the clinical subtype

Type 3		
0.54 (0.19–1.50)	Type 2	
**6.88 (3.79–12.49)**	**11.53 (3.46–38.49)**	Type 1

Odds ratio with 95% confidence intervals in parentheses. The comparison between the clinical subtypes should be read from left to right by comparing columns with rows. Bold values indicate a statistically significant difference.

### Pathological risk factors of LNM

Eighteen studies reported the pathological risk factors of LNM. Tumor size >10 mm (OR 4.18; 95% CI 1.91–9.17), tumor invasion into MP or deeper (OR 11.21; 95% CI 3.50–35.92), G2/G3 (OR 5.96; 95% CI 2.65–13.40), and LVI (OR 34.50; 95% CI 6.70–177.51) were significantly associated with LNM (Table 5; Fig. S1). No significant heterogeneity across the studies was found in any factor. The results of analyses for pathological risk factors in type 1 and 3 gNETs, separately, are presented in Table S3. Since it was difficult to analyze pathological risk factors in type 2 gNETs due to the very small numbers of cases, details of clinicopathological features of type 2 gNETs in each study are summarized in Table S4. Furthermore, we conducted the same analysis exclusively on SR cases as a sensitivity analysis (Table S5). Similar results were obtained regarding the tumor size and tumor invasion. However, the result of WHO grading did not reach statistical significance, and the risk of LVI could not be assessed due to the small sample size.

**Table 5:** Pathological risk factors of lymph node metastasis (LNM) in gastric neuroendocrine tumors

	No. of studies	No. of cases	No. of LNM	Pooled OR (95% CI)	*P*‐value	*P* for Q test	*I* ^*2*^	*P* for Egger's test^†^
Tumor size								
≤10 mm	–	350	6	Reference	–	–	–	–
>10 mm	11	231	51	4.18 (1.91–9.17)	<0.001	0.654	0	0.973
Tumor depth								
Mucosa/SM	–	377	13	Reference	–	–	–	–
MP or deeper	9	28	10	11.21 (3.50–35.92)	<0.001	0.578	14	–
WHO grading system								
G1	–	355	20	Reference	–	–	–	–
G2/G3	13	122	50	5.96 (2.65–13.40)	<0.001	0.101	41	0.556
LVI								
Negative	–	177	1	Reference	–	–	–	–
Positive	5	21	5	34.50 (6.70–177.51)	<0.001	0.917	0	–

^†^ Egger's test was used, if there were ≥10 studies in the meta‐analysis.

CI, confidence interval; G, grade; LVI, lymphovascular invasion; MP, muscularis propria; OR, odds ratio; SM, submucosa; WHO, World Health Organization.

### Assessment of publication bias

Visual inspection of funnel plots (Fig. S2) suggested a potential publication bias for the overall prevalence of LNM, which was also confirmed by Egger's test (*P* = 0.016). However, due to considerable heterogeneity, these results should be interpreted with caution. No publication bias was evident in the analysis of pathological risk factors for LNM (Table 5).

## DISCUSSION

Given that ER is often preferred for small gNETs,^20^, ^21^, ^23^ identifying risk factors for LNM in gNETs is crucial. Despite numerous studies evaluating LNM and prognosis in gNETs, the precise prevalence and risk factors for LNM in gNETs remain unclear due to the limited number of cases included in these studies. This meta‐analysis demonstrated that the pooled prevalence rate of LNM in patients with gNETs was 11.8%, exhibiting considerable heterogeneity. Furthermore, it identified type 2 and 3 gNETs as being at high risk for LNM. Moreover, tumor size >10 mm, tumor invasion into MP or deeper, G2/G3, and LVI were significantly associated with LNM. Taken together, our systematic review provides significant insights into the risk of LNM in gNETs. Based on these findings, we recommend considering SR in cases with identified risk factors.

We identified two clinical implications in this systematic review. First, type 2 and type 3 gNETs exhibited a high prevalence rate of LNM. Type 1 and type 2 gNETs, characterized as ECLomas, i.e., NETs consisting of ECL cells, due to chronic hypergastrinemia,^8^, ^45^ have generally been considered as having an association with a better prognosis compared to type 3.^46^, ^47^ However, in particular, data on type 2 gNETs had been scarce due to their rarity, and the guidelines' recommendation for this type of gNET was not based on data. In our meta‐analysis, the prevalence rate of LNM in type 1 gNETs was low (6.0%); in contrast, those in type 2 and type 3 gNETs were high (38.5% and 23.2%, respectively, for all cases; 26.8% and 29.1%, respectively, when limited to SR cases). Furthermore, both type 2 and type 3 gNETs exhibited a significantly higher LNM risk compared to type 1. However, no significant difference was found in the LNM risk between type 2 and type 3 gNETs. These results support the current guidelines' recommendation that SR for type 3 gNETs may be acceptable due to its high rate of LNM.^48^, ^49^ In contrast, our results do not support the guidelines' recommendation that local or limited excision can be recommended for type 2 gNETs. Based on our results, SR, rather than ER, might be recommended as an initial treatment for type 2 gNETs. However, it should be noted that data on type 2 gNETs are still not enough, and further accumulation of data is required for strong recommendation of treatment strategies in this type of gNETs.

Second, we were the first to unveil the detailed association between pathological risk factors and LNM in gNETs. To date, only one systematic review regarding type 1 gNETs has evaluated this association.^50^ In the systematic review, tumor size >10 mm and tumor invasion into MP were identified as predictors of LNM. However, the WHO grading system was not associated with LNM, possibly due to the limited number of cases, and LVI was not evaluated. Our systematic review, which encompassed all types of gNETs, identified several risk factors associated with LNM. These included G2/G3 grading, LVI, tumor size >10 mm, and tumor invasion into the MP or deeper layers. Furthermore, with the highest OR, LVI emerges as potentially the most crucial factor for LNM in gNETs; an accurate analysis of LVI, however, was not feasible when limited to SR cases. These factors were also identified as risk factors for metastasis in NETs occurring in organs other than the stomach.^51^, ^52^, ^53^ Our findings could assist clinicians in determining treatment strategies, especially after ER, i.e. additional SR or no additional treatment.

Although we demonstrated the significance of the clinical subtype and pathological risk factors for LNM, we cannot dismiss the possibility of confounding effects between them. Most pathological risk factors for LNM assessed were still significant in the analyses limited to type 1 and type 3 gNETs, respectively (Table S3), but this analysis could not be performed in type 2 gNETs due to the small number of cases. Furthermore, to avoid confounding, multivariate analyses are required. Considering the rarity of gNETs, it may be difficult to perform such an analysis including all clinical types and pathological factors, even in a large‐scale multicenter study. Further accumulation of data is required to overcome this limitation.

Wide variability was observed in the prevalence rate of LNM in gNETs across studies. This may be explained by two potential reasons. First, the prevalence rates of LNM varied among the clinical subtypes, and these rates differed across studies. Furthermore, the analysis in type 3 gNETs still exhibited substantial heterogeneity, with one study showing a very high rate of LNM contributing to this heterogeneity. In this study, all type 3 gNETs (14 cases) invaded MP or deeper, which may have led to a high rate of LNM. Second, the prevalence rates of LNM differed significantly between ER and SR cases, with studies including numerous ER cases yielding low prevalence rates of LNM. Indeed, only one case reported by Min *et al*. (2018) developed perigastric LNM 68 months after ER. Although LNM in ER cases were not necessarily reflected at the time of treatment and could be underestimated, as they were not evaluated pathologically, given that ER alone is generally performed for small and low‐risk gNETs, this heterogeneity may be understandable. Furthermore, the absence of heterogeneity in the four pathological risk factors suggests that pathological features may greatly affect the risk of LNM. Conversely, the impact of differences in treatment methods on LNM risk may be limited.

This study has several strengths. First, it is the first systematic review that pooled all types of gNETs. Second, we performed a comprehensive and systematic literature search with well‐defined inclusion criteria, carefully excluding redundant studies and including good‐quality studies based on the PRISMA guidelines. Third, subgroup and sensitivity analyses were performed to evaluate the stability of findings and identify potential factors responsible for inconsistencies.

There are also several limitations in this study. First, significant heterogeneity was observed in the summary estimate of the prevalence of LNM in gNETs, as described above. Thus, caution is required for interpreting the pooled estimate. Second, most studies included in this systematic review were retrospective. Third, in the ER cases, LNM was evaluated radiologically during the follow‐up instead of pathologically. This approach raises the possibility of hidden LNM being present in the ER report, especially in cases with a short follow‐up duration. In fact, a case of LNM recurrence after ER showed LNM development 68 months later. This case may suggest the possibility of late recurrence after ER in gNETs and highlight the necessity of long‐term follow‐up to accurately assess LNM. Furthermore, cases having LNM during the follow‐up after ER may not necessarily represent LNM at the time of ER treatment. Results in the analyses limited to SR cases were consistent with those in the main analyses in the clinical subtype, tumor size, and tumor depth. However, the risk of the WHO grading system for LNM did not reach statistical significance and the risk of LVI could not be assessed due to the small sample size when limited to SR cases; thus, further studies are required, especially for these factors. Lastly, most studies included in the analysis only provided univariate analysis for risk factors of LNM in gNETs. Nevertheless, it is noteworthy that this systematic review identified four pathological risk factors for LNM from the pooled data in gNETs, although some studies have reported the pathological risk factors.^38^, ^44^

In conclusion, the pooled prevalence rate of LNM in patients with gNET was 11.8%, but with significant heterogeneity. Our systematic review initially identified that type 2 gNETs, as well as type 3, were at high risk for LNM. Besides, tumor size of >10 mm, tumor invasion into MP or deeper, G2/G3, and LVI were pathological risk factors for LNM, and LVI was the most important risk factor. These findings could assist in determining the treatment strategy for gNETs. However, further accumulation of data would be required for a strong recommendation of treatment strategy, especially in type 2 gNETs.

## CONFLICT OF INTEREST

Authors declare no conflict of interest for this article.

## FUNDING INFORMATION

None.

## Supporting information


**Appendix S1** Literature research.
**Appendix S2** PRISMA 2020 main checklist.
**Table S1** Detailed characteristics of eligible studies.
**Table S2** Quality assessment of included studies using the Joanna Briggs Institute (JBI) critical appraisal tools for JBI systematic reviews.
**Table S3** Pathological risk factors of lymph node metastasis (LNM) in gastric neuroendocrine tumors (gNETs) according to the clinical subtype.
**Table S4** Details of clinicopathological features of type 2 gastric neuroendocrine tumors (gNETs) according to each study.
**Table S5** Pathological risk factors of lymph node metastasis (LNM) in gastric neuroendocrine tumors (gNETs) limited to the surgical resection (SR) cases.
**Figure S1** Forest plot depicting pathological risk factors for lymph node metastasis (LNM) in gastric neuroendocrine tumors (gNETs).
**Figure S2** Funnel plots in the analysis of the overall prevalence of lymph node metastasis (LNM).
