# Supplementary material for: Type 2 and type 3 gastric neuroendocrine tumors have high risk of lymph node metastasis: Systematic review and meta‐analysis
Source: Dig Endosc. 2025 Apr 1;37(8):834–43. doi: 10.1111/den.15026 (PMC12333324; doi:10.1111/den.15026)
Supplement: Supplementary file 1 — Appendix S1 Literature research. Appendix S2 PRISMA 2020 main checklist. Table S1 Detailed characteristics of eligible studies. Table S2 Quality assessment of included studies using the Joanna Briggs Institute (JBI) critical appraisal tools for JBI systematic reviews. Table S3 Pathological risk factors of lymph node metastasis (LNM) in gastric neuroendocrine tumors (gNETs) according to the clinical subtype. Table S4 Details of clinicopathological features of type 2 gastric neuroendocrine tumors (gNETs) according to each study. Table S5 Pathological risk factors of lymph node metastasis (LNM) in gastric neuroendocrine tumors (gNETs) limited to the surgical resection (SR) cases. Figure S1 Forest plot depicting pathological risk factors for lymph node metastasis (LNM) in gastric neuroendocrine tumors (gNETs). Figure S2 Funnel plots in the analysis of the overall prevalence of lymph node metastasis (LNM). [file DEN-37-834-s001.docx]

**Supplementary files**

**Appendix S1.** Literature research

**Database:**

**EBM Reviews - Cochrane Central Register of Controlled Trials <May 2023>, EBM Reviews - Cochrane Database of Systematic Reviews <2005 to June 6, 2023>, Embase <1974 to 2023 June 09>, OVID Medline Epub Ahead of Print, In-Process & Other Non-Indexed Citations, Ovid MEDLINE(R) Daily and Ovid MEDLINE(R) 1946 to Present**

**Search Strategy:**
**1**  exp Neuroendocrine Tumors/ or exp neuroendocrine tumor/ (373231)
**2**  (neuroendocrine adj3 (tumor* or tumour* or carcinoma* or cancer* or malignan* or neoplas*)).tw,kw. (78201)
**3**  carcinoid*.tw,kw. (40348)
**4**  (APUD adj3 (tumor* or tumour* or carcinoma* or cancer* or malignan* or neoplas*)).tw,kw. (259)
**5**  (NETs or Mixed adenoneuroendocrine carcinoma* or MANEC or apudoma*).tw,kw. (44472)
**6**  or/1-5 (433843)
**7**  exp Gastrointestinal Tract/ (786704)
**8**  (stomach or gastric or gastro* or intragastric or duoden* or esophag* or oesophag* or intestin* or enteral or enteric or cecum or cecal or ileum or ileal or ileocecal or jejunum or jejunal).tw,kw. (3194159)
**9**  (colorectum or colon or rectum or rectal or small bowel or large bowel).tw,kw. (851140)
**10**  (GI tract or UGI tract or LGI tract or digestive tract or alimentary tract or Gut).tw,kw. (404644)
**11**  or/7-10 (4179498)
**12**  6 and 11 (55665)
**13**  exp Lymph Nodes/ (307156)
**14**  Lymph*.tw,kw. (2558263)
**15**  (LN or LNs).tw,kw. (76409)
**16**  exp neoplasm metastasis/ (1016512)
**17**  (metastasis or metastases).tw,kw. (1074906)
**18**  or/13-17 (3757450)
**19**  12 and 18 (21538)
**20**  conference abstract.pt. or Congresses as Topic/ or Conference Review.pt. (4927703)
**21**  19 not 20 (16875)
**22**  limit 21 to english language [Limit not valid in CDSR; records were retained] (14173)
**23**  (exp animals/ or exp animal/ or exp nonhuman/ or exp animal experiment/ or animal model/ or animal tissue/ or non human/) not (humans/ or human/ or human experiment/) (12551191)
**24**  22 not 23 use ppez,oemezd (13819)
**25**  (rat or rats or mice or mouse or swine or porcine or murine or sheep or lambs or pigs or piglets or rabbit or rabbits or cat or cats or dog or dogs or cattle or bovine or monkey or monkeys or trout or marmoset$1).ti. not (human* or men or women or patients or subjects or paticipants).tw. (3756471)
**26**  24 not 25 use ppez,oemezd (13807)
**27**  19 use coch,cctr (235)
**28**  26 or 27 (13813)

**29** limit 28 to yr="1990 -Current" (11212)

**30** remove duplicates from 29 (7769)

**Appendix S2.** PRISMA 2020 Main Checklist

| **Topic** | **No.** | **Item** | **Location where item is reported** |
| --- | --- | --- | --- |
| **TITLE** |  |  |  |
| **Title** | 1 | Identify the report as a systematic review. | p1 |
| **ABSTRACT** |  |  |  |
| **Abstract** | 2 | See the PRISMA 2020 for Abstracts checklist | P3,4 |
| **INTRODUCTION** |  |  |  |
| **Rationale** | 3 | Describe the rationale for the review in the context of existing knowledge. | P5,6 |
| **Objectives** | 4 | Provide an explicit statement of the objective(s) or question(s) the review addresses. | P6 |
| **METHODS** |  |  |  |
| **Eligibility criteria** | 5 | Specify the inclusion and exclusion criteria for the review and how studies were grouped for the syntheses. | P7,8 |
| **Information sources** | 6 | Specify all databases, registers, websites, organisations, reference lists and other sources searched or consulted to identify studies. Specify the date when each source was last searched or consulted. | P7 |
| **Search strategy** | 7 | Present the full search strategies for all databases, registers and websites, including any filters and limits used. | P7,  Supplementary content |
| **Selection process** | 8 | Specify the methods used to decide whether a study met the inclusion criteria of the review, including how many reviewers screened each record and each report retrieved, whether they worked independently, and if applicable, details of automation tools used in the process. | P7,8 |
| **Data collection process** | 9 | Specify the methods used to collect data from reports, including how many reviewers collected data from each report, whether they worked independently, any processes for obtaining or confirming data from study investigators, and if applicable, details of automation tools used in the process. | P7,8 |
| **Data items** | 10a | List and define all outcomes for which data were sought. Specify whether all results that were compatible with each outcome domain in each study were sought (e.g. for all measures, time points, analyses), and if not, the methods used to decide which results to collect. | P8,9 |
|  | 10b | List and define all other variables for which data were sought (e.g. participant and intervention characteristics, funding sources). Describe any assumptions made about any missing or unclear information. | P8,9 |
| **Study risk of bias assessment** | 11 | Specify the methods used to assess risk of bias in the included studies, including details of the tool(s) used, how many reviewers assessed each study and whether they worked independently, and if applicable, details of automation tools used in the process. | P9,10 |
| **Effect measures** | 12 | Specify for each outcome the effect measure(s) (e.g. risk ratio, mean difference) used in the synthesis or presentation of results. | p10 |
| **Synthesis methods** | 13a | Describe the processes used to decide which studies were eligible for each synthesis (e.g. tabulating the study intervention characteristics and comparing against the planned groups for each synthesis (item 5)). | p10,11 |
|  | 13b | Describe any methods required to prepare the data for presentation or synthesis, such as handling of missing summary statistics, or data conversions. | p10,11 |
|  | 13c | Describe any methods used to tabulate or visually display results of individual studies and syntheses. | p10,11 |
|  | 13d | Describe any methods used to synthesize results and provide a rationale for the choice(s). If meta-analysis was performed, describe the model(s), method(s) to identify the presence and extent of statistical heterogeneity, and software package(s) used. | p10,11 |
|  | 13e | Describe any methods used to explore possible causes of heterogeneity among study results (e.g. subgroup analysis, meta-regression). | p10,11 |
|  | 13f | Describe any sensitivity analyses conducted to assess robustness of the synthesized results. | p10,11 |
| **Reporting bias assessment** | 14 | Describe any methods used to assess risk of bias due to missing results in a synthesis (arising from reporting biases). | P9,10 |
| **Certainty assessment** | 15 | Describe any methods used to assess certainty (or confidence) in the body of evidence for an outcome. | p10,11 |
| **RESULTS** |  |  |  |
| **Study selection** | 16a | Describe the results of the search and selection process, from the number of records identified in the search to the number of studies included in the review, ideally using a flow diagram. | p12 |
|  | 16b | Cite studies that might appear to meet the inclusion criteria, but which were excluded, and explain why they were excluded. | P12, Fig. 1 |
| **Study characteristics** | 17 | Cite each included study and present its characteristics. | Table 1, Table S1 |
| **Risk of bias in studies** | 18 | Present assessments of risk of bias for each included study. | Table S2 |
| **Results of individual studies** | 19 | For all outcomes, present, for each study: (a) summary statistics for each group (where appropriate) and (b) an effect estimate and its precision (e.g. confidence/credible interval), ideally using structured tables or plots. | Fig. 2 |
| **Results of syntheses** | 20a | For each synthesis, briefly summarise the characteristics and risk of bias among contributing studies. | p13,14,15 |
|  | 20b | Present results of all statistical syntheses conducted. If meta-analysis was done, present for each the summary estimate and its precision (e.g. confidence/credible interval) and measures of statistical heterogeneity. If comparing groups, describe the direction of the effect. | p13,14,15 |
|  | 20c | Present results of all investigations of possible causes of heterogeneity among study results. | p13,14,15 |
|  | 20d | Present results of all sensitivity analyses conducted to assess the robustness of the synthesized results. | p13,14,15 |
| **Reporting biases** | 21 | Present assessments of risk of bias due to missing results (arising from reporting biases) for each synthesis assessed. | p15 |
| **Certainty of evidence** | 22 | Present assessments of certainty (or confidence) in the body of evidence for each outcome assessed. | p13,14,15 |
| **DISCUSSION** |  |  |  |
| **Discussion** | 23a | Provide a general interpretation of the results in the context of other evidence. | p16,17,18,19 |
|  | 23b | Discuss any limitations of the evidence included in the review. | P20 |
|  | 23c | Discuss any limitations of the review processes used. | P20 |
|  | 23d | Discuss implications of the results for practice, policy, and future research. | P17,18,19,20 |
| **OTHER INFORMATION** |  |  |  |
| **Registration and protocol** | 24a | Provide registration information for the review, including register name and registration number, or state that the review was not registered. | P7 |
|  | 24b | Indicate where the review protocol can be accessed, or state that a protocol was not prepared. | P7 |
|  | 24c | Describe and explain any amendments to information provided at registration or in the protocol. | P7 |
| **Support** | 25 | Describe sources of financial or non-financial support for the review, and the role of the funders or sponsors in the review. | P22 |
| **Competing interests** | 26 | Declare any competing interests of review authors. | P22 |
| **Availability of data, code and other materials** | 27 | Report which of the following are publicly available and where they can be found: template data collection forms; data extracted from included studies; data used for all analyses; analytic code; any other materials used in the review. | Table 1, Table S1 |

*From:* Page MJ, McKenzie JE, Bossuyt PM, Boutron I, Hoffmann TC, Mulrow CD, et al. The PRISMA 2020 statement: an updated guideline for reporting systematic reviews. MetaArXiv. 2020, September 14. DOI: 10.31222/osf.io/v7gm2. For more information, visit: [www.prisma-statement.org](file:///C:\Users\owner\AppData\Local\Microsoft\Windows\INetCache\Content.Outlook\4F9ICXIH\www.prisma-statement.org)

**Supplementary Tables**

**Table S1.** The detailed characteristics of eligible studies

| Authors  (year) | Country | Setting | Study design | Enrollment  time period | Type of  treatment | No. of  patients | LNM in surgery | LNM in all cases | 2 x 2 table calculation for OR | | | | Clinical subtype |  |
| --- | --- | --- | --- | --- | --- | --- | --- | --- | --- | --- | --- | --- | --- | --- |
|  |  |  |  |  |  |  |  |  | Tumor size  > 10mm | Tumor  depth of MP or deeper | Positive LVI | G2/G3 |  | Follow-up  Period (mos.) |
| Rindi G, et al.  (1995) | Italy | Multi-center | Retrospective cohort | NA | ER (12),  SR (43) | 45 | NA | 4 | NA | NA | NA | NA | Available | NA (type 1)  6 yrs (type 2)  4 yrs (type 3) |
| Li QL, et al.  (2012) | China | Single-center | Retrospective cohort | 2008–2010 | ER(18), SR(1)^‡^ | 19 | 0 | 0 | Available | Available | Available | Available | Available | 24.4 |
| Merola E, et al.  (2012) | Italy | Single-center | Prospective cohort | 1993–2008 | ER (32),  SR (1)^‡^ | 33 | 0 | 0 | NA | Available | NA | NA | Available  (all, type 3) | 46 |
| Endo S, et al.  (2012) | Japan | Multi-center | Retrospective cohort | 1998–2011 | ER (3),  SR (13) | 16^§^ | 2 | 2 | Available | Available | NA | Available | Available | 53 |
| Uygun A, et al.  (2014) | Turkey | Single-center | Prospective cohort | 1999–2012 | ER (22) | 22 | NA | 0 | Available | NA | NA | NA | Available  (all, type 1) | 7 yrs |
| Jung HJ, et al.  (2015) | Korea | Single-center | Retrospective cohort | 2002–2012 | ER (18),  SR (8) | 26 | 0 | 0 | NA | NA | Available | NA | NA | 30 (ER case)  50 (SR case) |
| Shen C, et al.  (2016) | China | Single-center | Retrospective cohort | 2009–2015 | ER (25),  SR (110) | 46 | NA | 7 | NA | NA | NA | Available | NA | 22 |
| Lee HE, et al.  (2016) | US | Single-center | Retrospective cohort | 1994–2015 | ER (16),  SR (29) | 45 | 11 | 11 | NA | NA | NA | NA | Available | 14 |
| Sagatun L, et al.  (2016) | Norway | Single-center | Retrospective cohort | 1993–2013 | SR (8) | 8^§^ | 3 | 3 | NA | Available | NA | Available | NA | 52.5 |
| Xu TM, et al.  (2016) | China | Single-center | Retrospective cohort | 1995–2015 | SR (3)^¶^ | 3 | 0 | 0 | NA | NA | NA | NA | Available | NA |
| Chung CS, et al.  (2018) | Taiwan | Multi-center | Retrospective cohort | 2010–2016 | ER (57),  SR (55), Unknown (75) | 187 | NA | 24 | Available | NA | NA | Available | NA | 2.5 yrs |
| Min BH, et al.  (2018) | Korea | Single-center | Retrospective cohort | 2005–2014 | ER (17),  SR (15)^‡^ | 32 | 2 | 3 | NA | NA | NA | Available | Available  (all, type 3) | 59 (ER case)  70 (SR case) |
| Vanoli A, et al.  (2018) | Italy | Multi-center | Retrospective cohort | 1980–2017 | ER (87),  SR (70) | 157 | NA | 16 | NA | NA | NA | NA | Available | 93 |
| Daskalakis K, et al.  (2019) | Greece | Single-center | Retrospective cohort | 1997–2017 | ER (97),  SR (17) | 114 | NA | 3 | NA | NA | NA | NA | Available  (all, type 1) | 45.3 |
| Crown A, et al.  (2019) | US | Multi-center | Retrospective cohort | 2000–2016 | ER (25),  SR (50) | 75 | NA | 12 | NA | NA | NA | NA | Available | NA |
| Chen X, et al.  (2019) | China | Single-center | Retrospective cohort | 2011–2018 | ER (24) | 24 | NA | 0 | Available | Available | Available | Available | NA | NA |
| Chen Y, et al.  (2020) | China | Multi-center | Prospective and retrospective cohort | 2012–2019 | ER (136) | 136 | NA | 0 | Available | Available | NA | Available | Available  (all, type 1) | 22 |
| Trinh VQ, et al.  (2020) | US | Multi-center | Retrospective cohort | 2002–2019 | SR (66) | 66 | 12 | 12 | NA | NA | NA | NA | Available  (all, type 3) | 49 |
| Chin JL, et al.  (2021) | Ireland | Multi-center | Retrospective cohort | 2005–2017 | ER (12),  SR (7) | 19^§^ | NA | 3 | NA | NA | NA | NA | Available  (all, type 1) | 52 |
| Exarchou K, et al.  (2021) | UK | Multi-center | Retrospective cohort | 2006–2019 | ER (10),  SR (26) | 36^§^ | 11 | 11 | Available | NA | NA | NA | Available  (all type 3) | 56 |
| Hirasawa T, et al.  (2021) | Japan | Multi-center | Retrospective cohort | 1987–2015 | ER (48),  SR (96)^‡^ | 144 | 15 | 15 | Available | Available | NA | Available | Available  (all, type 3) | 32 (ER case)  51 (SR case) |
| Li X, et al.  (2021) | China | Single-center | Retrospective cohort | 2012–2019 | SR (94) | 94 | 68 | 68 | NA | NA | NA | Available | NA | NA |
| Exarchou K, et al.  (2022) | UK | Multi-center | Retrospective cohort | 2003–2019 | ER (10),  SR (8) | 18^§^ | 1 | 1 | Available | NA | NA | Available | Available  (all, type 1) | 66 |
| Kurtulan O, et al.  (2022) | Turkey | Multi-center | Retrospective cohort | 2000–2015 | SR (44) | 44 | 15 | 15 | NA | NA | NA | NA | Available | NA |
| Ryu DG, et al.  (2022) | Korea | Single-center | Retrospective cohort | 2008–2020 | ER (14),  SR (4) | 18^§^ | 2 | 2 | NA | Available | Available | Available | Available | 56.2 |
| Sekar A, et al.  (2022) | India | Single-center | Retrospective cohort | 2011–2020 | Unknown (65) | 65 | NA | 18 | NA | NA | NA | Available | Available | NA |
| Kim Y, et al.  (2023) | Korea | Single-center | Retrospective cohort | 2000–2020 | ER (116),  SR (23) | 139 | 6 | 6 | NA | NA | NA | NA | NA | 36 |
| Namikawa K, et al. (2023) | Japan | Multi-center | Retrospective cohort | 1991–2019 | ER (84),  SR (27)^‡^ | 111^§^ | 4 | 4 | Available | Available | Available | Available | Available  (all type 1) | 10.1 yrs |

† Including 99 cases with NECs, MiNEN, and gastrinomas in the number of treatments, which were excluded from this meta-analysis; 10 in the report by Rindi et al. and 89 cases in the report by Shen et al.

‡ Including 25 cases with additional gastrectomy; one in the report by Li et al., one in the report by Merola et al., five in the report by Min et al., 15 cases in the report by Hirasawa et al., and three in the report by Namikawa et al.

§ There were some cases with other treatments in each report, which were not included in this meta-analysis: three cases with no treatment in the report by Endo et al., 18 cases with no resection by Sagatun et al., 30 cases with no treatment by Chin et al., nine cases with no resection by Exarchou et al. (2021), 87 cases with no treatment by Exarchou et al. (2022), four cases with no treatment by Ryu et al., 61 cases with no treatment by Namikawa et al.

¶ Only patients with type 2 gNETs were included in the analysis, given the clearly stated type of treatment.

OR, odds ratio; LNM, lymph node metastasis; MP, muscularis propria; LVI, lymphovascular invasion; G, grade; NA, not applicable; ER, endoscopic resection; SR, surgical resection; US, United States; UK, United Kingdom; NECs, neuroendocrine carcinomas; MiNEN, Mixed neuroendocrine-non-neuroendocrine neoplasia.

**Table S2.** Quality assessment of included studies using the JBI Critical Appraisal Tools for JBI Systematic Reviews.

|  | 1. Was the sample frame appropriate to address the target population? | 2. Were study participants sampled in an appropriate way? | 3. Was the sample size adequate? | 4. Were the study subjects and the setting described in detail? | 5. Was the data analysis conducted with sufficient coverage of the identified sample? | 6. Were valid methods used for the identification of the condition? | 7. Was the condition measured in a standard, reliable way for all participants? | 8. Was there appropriate statistical analysis? | 9. Was the response rate adequate, and if not, was the low response rate managed appropriately? | Risk of bias | Overall appraisal |
| --- | --- | --- | --- | --- | --- | --- | --- | --- | --- | --- | --- |
| Rindi G, et al.  (1995) | Yes | Yes | NA | Yes | Yes | Yes | Yes | Yes | NA | Low | Include |
| Li QL, et al.  (2012) | Yes | Yes | NA | Yes | Yes | Yes | Yes | Yes | NA | Low | Include |
| Merola E, et al.  (2012) | Yes | Yes | NA | Yes | Yes | Yes | Yes | Yes | NA | Low | Include |
| Endo S, et al.  (2012) | Yes | Yes | NA | Yes | Yes | Yes | Yes | Yes | NA | Low | Include |
| Uygun A, et al.  (2014) | Yes | Yes | NA | Yes | Yes | Yes | Yes | Yes | NA | Low | Include |
| Jung HJ, et al.  (2015) | Yes | Yes | NA | Yes | Yes | Yes | Yes | Yes | NA | Low | Include |
| Shen C, et al.  (2016) | Yes | Yes | NA | Yes | Yes | Yes | Yes | Yes | NA | Low | Include |
| Lee HE, et al.  (2016) | Yes | Yes | NA | Yes | Yes | Yes | Yes | Yes | NA | Low | Include |
| Sagatun L, et al.  (2016) | Yes | Yes | NA | Yes | Yes | Yes | Yes | Yes | NA | Low | Include |
| Xu TM, et al.  (2016) | Yes | Yes | NA | Yes | Yes | Yes | Yes | Yes | NA | Low | Include |
| Chung CS, et al.  (2018) | Yes | Yes | NA | Unclear | Yes | Yes | Yes | Yes | NA | Moderate | Include |
| Min BH, et al.  (2018) | Yes | Yes | NA | Yes | Yes | Yes | Yes | Yes | NA | Low | Include |
| Vanoli A, et al.  (2018) | Yes | Yes | NA | Yes | Yes | Yes | Yes | Yes | NA | Low | Include |
| Daskalakis K, et al.  (2019) | Yes | Yes | NA | Yes | Yes | Yes | Yes | Yes | NA | Low | Include |
| Crown A, et al.  (2019) | Yes | Yes | NA | Yes | Yes | Unclear | Yes | Yes | NA | Moderate | Include |
| Chen X, et al.  (2019) | Yes | Yes | NA | Yes | Yes | Unclear | Yes | Yes | NA | Moderate | Include |
| Chen Y, et al.  (2020) | Yes | Yes | NA | Yes | Yes | Yes | Yes | Yes | NA | Low | Include |
| Trinh VQ, et al.  (2020) | Yes | Yes | NA | Yes | Yes | Yes | Yes | Yes | NA | Low | Include |
| Chin JL, et al.  (2021) | Yes | Yes | NA | Yes | Yes | Yes | Yes | Yes | NA | Low | Include |
| Exarchou K, et al.  (2021) | Yes | Yes | NA | Yes | Yes | Yes | Yes | Yes | NA | Low | Include |
| Hirasawa T, et al.  (2021) | Yes | Yes | NA | Yes | Yes | Yes | Yes | Yes | NA | Low | Include |
| Li X, et al.  (2021) | Yes | Yes | NA | Yes | Yes | Yes | Yes | Yes | NA | Low | Include |
| Exarchou K, et al.  (2022) | Yes | Yes | NA | Yes | Yes | Yes | Yes | Yes | NA | Low | Include |
| Kurtulan O, et al.  (2022) | Yes | Yes | NA | Yes | Yes | Yes | Yes | Yes | NA | Low | Include |
| Ryu DG, et al.  (2022) | Yes | Yes | NA | Yes | Yes | Yes | Yes | Yes | NA | Low | Include |
| Sekar A, et al.  (2022) | Yes | Yes | NA | Unclear | Yes | Unclear | Yes | Yes | NA | Moderate | Include |
| Kim Y, et al.  (2023) | Yes | Yes | NA | Yes | Yes | Yes | Yes | Yes | NA | Low | Include |
| Namikawa K, et al.  (2023) | Yes | Yes | NA | Yes | Yes | Yes | Yes | Yes | NA | Low | Include |

JBI, Joanna Briggs Institute; NA, not applicable

**Table S3.** Pathological risk factors of LNM in gNETs according to the clinical subtype.

|  |  | No. of  studies | No. of  cases | No. of  LNM | Pooled OR,  (95% CI) | *p* | *p* for Q test | *I^2^* | *p* for  Egger’s test^†^ |
| --- | --- | --- | --- | --- | --- | --- | --- | --- | --- |
| **Type 1** |  |  |  |  |  |  |  |  |  |
| **Tumor size** | ≤ 10 mm |  | 209 | 2 | Reference |  |  |  |  |
|  | > 10 mm | 6 | 65 | 3 | 4.17 (1.08–16.15) | 0.039 | 0.958 | 0 | – |
| **Tumor depth** | Mucosa/SM |  | 224 | 4 | Reference |  |  |  |  |
|  | MP or deeper | 5 | 2 | 0 | 16.02 (2.34–109.77) | 0.005 | 0.800 | 0 | – |
| **WHO grading**  **system** | G1 |  | 172 | 3 | Reference |  |  |  |  |
|  | G2/G3 | 6 | 61 | 0 | 1.25 (0.26–5.89) | 0.779 | 0.741 | 0 | – |
| **LVI** | Negative |  | 99 | 1 | Reference |  |  |  |  |
|  | Positive | 2 | 19 | 3 | 16.66 (2.05–135.57) | 0.009 | 0.963 | 0 | – |
| **Type 2^‡^** |  |  |  |  |  |  |  |  |  |
| **Type 3^§^** |  |  |  |  |  |  |  |  |  |
| **Tumor size** | ≤ 10 mm |  | 56 | 4 | Reference |  |  |  |  |
|  | > 10 mm | 4 | 83 | 24 | 3.13 (1.10–8.92) | 0.032 | 0.647 | 0 | – |
| **Tumor depth** | Mucosa/SM |  | 129 | 9 | Reference |  |  |  |  |
|  | MP or deeper | 5 | 23 | 10 | 8.26 (2.20–30.99) | 0.002 | 0.584 | 0 | – |
| **WHO grading**  **system** | G1 |  | 92 | 5 | Reference |  |  |  |  |
|  | G2/G3 | 5 | 59 | 16 | 4.21 (1.60–11.08) | 0.004 | 0.731 | 0 | – |

† Egger’s test was used, if there were ≥ 10 studies in the meta-analysis.

‡ All pathological factors could not be analyzed due to the limited data. The details of each study were presented in Table S4.

§ LVI could not be analyzed because only one study was available.

LNM, lymph node metastasis; gNETs, gastric neuroendocrine tumors; OR, odds ratio; CI, confidence interval; SM, submucosa; MP, muscularis propria; G, grade; WHO, World Health Organization; LVI, lymphovascular invasion.

**Table S4.** Details of clinicopathological features of type 2 gNETs according to each study.

|  | Rindi G, et al | Lee HE, et al. | Xu TM, et al. | Crown A, et al. | Sekar A, et al |
| --- | --- | --- | --- | --- | --- |
| All cases | 7 | 6 | 3 | 4 | 8 |
| LNM | 2 | 2 | 0 | 1 | 6 |
| Treatment method |  |  |  |  |  |
| ER | NA | NA | 0 | 1 | NA |
| SR | NA | NA | 3 | 3 | NA |
| Pathological risk factor |  |  |  |  |  |
| Tumor size (cm) | 0.5 (0.1–1.6)^†^ | 2.0 (± 1.3)^‡^ | 2–5^§^ | 2.4^‡^ | NA |
| Tumor depth |  |  |  |  |  |
| Mucosa/SM | 6 | 5 | 3 | 2 | 5 |
| MP or deeper | 1 | 1 | 0 | 1 | 2 |
| WHO grading |  |  |  |  |  |
| G1 | NA | 2 | NA | NA | 4 |
| G2/G3 | NA | 4 | NA | NA | 4 |
| LVI |  |  |  |  |  |
| Negative | 5 | NA | NA | NA | NA |
| Positive | 2 | NA | NA | NA | NA |

† Median (range).

‡ Mean (± standard deviation)

§ Range.

gNETs, gastric neuroendocrine tumors; LNM, lymph node metastasis; ER, endoscopic resection; NA, no assessment; SR, surgical resection; SM, submucosa; MP, muscularis propria; WHO, World Health Organization; G, grade; LVI, lymphovascular invasion.

**Table S5.** Pathological risk factors of LNM in gNETs limited to the SR cases.

|  |  | No. of  studies | No. of  cases | No. of  LNM | Pooled OR,  (95% CI) | *p* | *p* for Q test | *I^2^* | *p* for  Egger’s test^†^ |
| --- | --- | --- | --- | --- | --- | --- | --- | --- | --- |
| Tumor size | ≤ 10 mm |  | 60 | 4 | Reference |  |  |  |  |
|  | > 10 mm | 6 | 88 | 25 | 2.96 (1.10–7.92) | 0.031 | 0.560 | 0 | – |
| Tumor depth | Mucosa/SM |  | 90 | 9 | Reference |  |  |  |  |
|  | MP or deeper | 4 | 20 | 8 | 5.08 (1.71–15.11) | 0.004 | 0.713 | 0 | – |
| WHO grading system | G1 |  | 66 | 11 | Reference |  |  |  |  |
|  | G2/G3 | 5 | 137 | 75 | 2.14 (0.91–5.02) | 0.081 | 0.764 | 0 | – |
| LVI^‡^ | Negative |  | 9 | 0 | Reference |  |  |  |  |
|  | Positive | 2 | 0 | 0 | – | – | – | – | – |

† Egger’s test was used, if there were ≥ 10 studies in the meta-analysis.

‡ LVI could not be analyzed due to the limited data.

LNM, lymph node metastasis; gNETs, gastric neuroendocrine tumors; SR, surgical resection; OR, odds ratio; CI, confidence interval; SM, submucosa; MP, muscularis propria; G, grade; WHO, World Health Organization; LVI, lymphovascular invasion.

**Supplementary Figures**

**
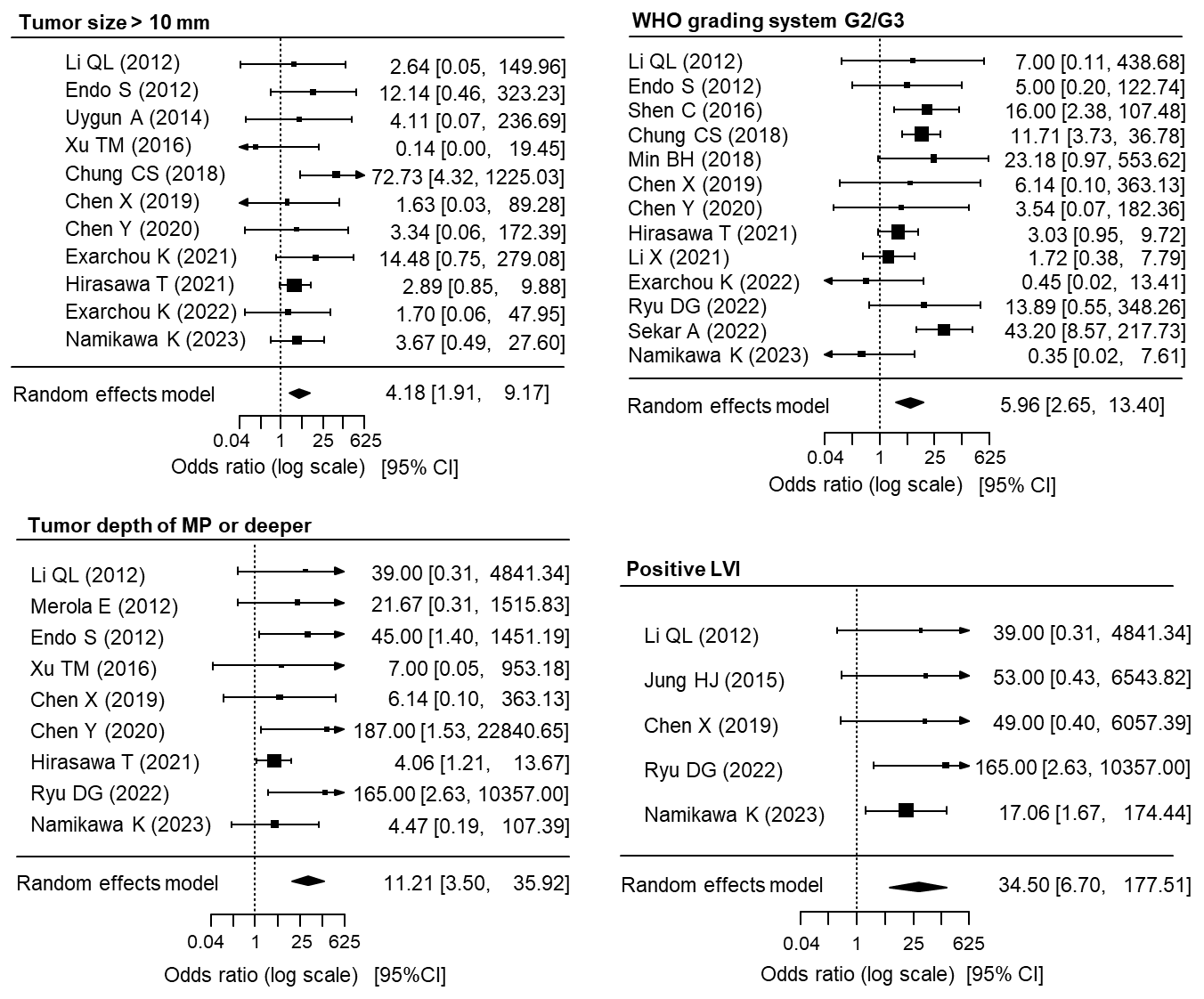
**

**Fig. S1.** Forest plot depicting pathological risk factors for LNM in gNETs.

LNM, lymph node metastasis; gNETs, gastric neuroendocrine tumors; MP, muscularis propria; LVI, lymphovascular invasion; CI, confidence interval.

**
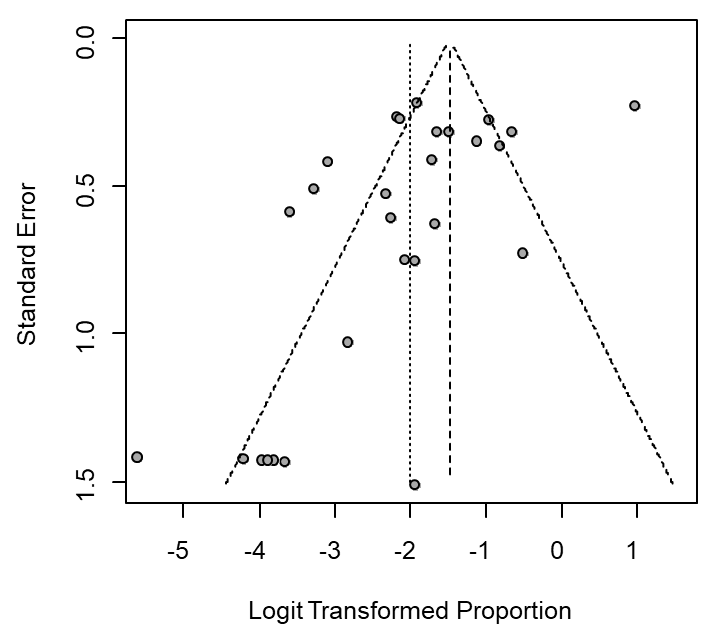
Fig. S2.** Funnel plots in the analysis of the overall prevalence of LNM

LNM, lymph node metastasis.
